# FANCJ suppresses microsatellite instability and lymphomagenesis independent of the Fanconi anemia pathway

**DOI:** 10.1101/gad.272740.115

**Published:** 2015-12-15

**Authors:** Kenichiro Matsuzaki, Valerie Borel, Carrie A. Adelman, Detlev Schindler, Simon J. Boulton

**Affiliations:** 1 DNA Damage Response Laboratory, Clare Hall Laboratories, The Francis Crick Institute, South Mimms EN6 3LD, United Kingdom;; 2 Department of Human Genetics, Biozentrum, University of Wurzburg, 97074 Wurzburg, Germany

**Keywords:** DNA repair, FANCJ, Fanconi anemia, genome stability, microsatellite instability

## Abstract

Microsatellites are short tandem repeat sequences that are highly prone to expansion/contraction due to their propensity to form non-B-form DNA structures, which hinder DNA polymerases and provoke template slippage. Although error correction by mismatch repair plays a key role in preventing microsatellite instability (MSI), which is a hallmark of Lynch syndrome, activities must also exist that unwind secondary structures to facilitate replication fidelity. Here, we report that *Fancj* helicase-deficient mice, while phenotypically resembling Fanconi anemia (FA), are also hypersensitive to replication inhibitors and predisposed to lymphoma. Whereas metabolism of G4-DNA structures is largely unaffected in *Fancj*^−/−^ mice, high levels of spontaneous MSI occur, which is exacerbated by replication inhibition. In contrast, MSI is not observed in *Fancd2*^−/−^ mice but is prevalent in human FA-J patients. Together, these data implicate FANCJ as a key factor required to counteract MSI, which is functionally distinct from its role in the FA pathway.

## Abstract (precis)

Matsuzaki et al. generated and phenotypically characterized mice deficient for FANCJ and established that FANCJ functions either downstream from FANCD2 or in a parallel repair pathway. The findings presented support a model in which FANCJ counteracts the formation of secondary structures that arise during replication of microsatellite sequences, which minimizes the potential for strand slippage during DNA polymerization, thus implicating FANCJ as a key factor in human diseases.

Maintenance of genome integrity during DNA replication is of vital importance to ensure that daughter cells inherit an intact copy of the genetic code. Repetitive DNA sequences are a particular challenge to genome stability due to their propensity to form secondary structures within template or nascent DNA strands that hinder replisome progression and promote template slippage. Microsatellites are repetitive sequences of 1–10 base pairs (bp) of DNA (Boyer et al. 2013). The expansion or contraction of microsatellites during DNA replication has been implicated in a wide range of genetic disorders, including neuromuscular or neurological diseases and cancer. A link between human disease and repetitive sequence instability is most clearly illustrated for trinucleotide repeats (Mirkin 2007), whose expansion is a recurrent cause of Friedreich's ataxia (GAA/TTC repeats) and Huntington's disease (CAG/CTG repeats). Microsatellite instability (MSI) is also a hallmark of Lynch syndrome-associated cancers (Aaltonen et al. 1993; Boland and Goel 2010), which are caused by mutations in DNA mismatch repair (MMR) genes. Although MMR deficiency is associated with hereditary and sporadic colorectal cancers in humans, MMR-deficient mice are primarily predisposed to lymphoma (Li 2008). Current evidence suggests that MMR corrects slipped strand mispairing resulting from additions or deletions in the newly synthesized strand, which arise during secondary structure-triggered template slippage or when the replication of the repeats is impaired. Given the strong association with human disease, understanding the mechanisms that maintain the integrity of repetitive sequences is of great clinical importance.

While mechanisms exist to directly detect structural alterations in DNA, including helix-distorting lesions and base–base mismatches, other lesions may go undetected until encountered by the DNA replication machinery. When this occurs, repair must be orchestrated in the context of the replication fork, necessitating coordination of checkpoint, repair, and replication factors. In response to replication fork blockages such as interstrand cross-links (ICLs), the ATR-dependent replication stress checkpoint, Fanconi anemia (FA), and homologous recombination (HR) pathways are essential for replication fork repair and restart (Clauson et al. 2013). Repair of ICLs requires nucleolytic processing, translesion DNA synthesis, and HR (Deans and West 2011). The requirement for HR stems from the generation of DNA double-strand breaks (DSBs) that arise from nucleolytic processing events. The FA pathway is comprised of at least 16 gene products defective in FA patients (Clauson et al. 2013), which present with progressive bone marrow failure, developmental abnormalities, subfertility, and tumor predisposition (Alter and Kupfer 2002; Alter 2003; Crossan and Patel 2012). At the molecular level, the primary function of the FA pathway appears to be to induce monoubiquitylation of the heterodimeric FANCD2/FANCI complex, which coordinates ICL incision and recruitment of downstream repair factors (for review, see Kim and D'Andrea 2012).

One of the most enigmatic FA proteins is FANCJ, a DEAH superfamily 2 helicase and part of the subfamily of Fe-S cluster-containing helicases, which also includes XPD, RTEL1, and CHL1 (Rudolf et al. 2006; Gari et al. 2012; Brosh and Cantor 2014). Biallelic mutations in FANCJ give rise to FA complementation group J (Levitus et al. 2005; Levran et al. 2005; Litman et al. 2005), whereas monoallelic mutations predispose to ovarian and breast cancers (Seal et al. 2006; Rafnar et al. 2011). More recently, a significant association with pancreatic and colorectal cancer was found (Rafnar et al. 2011). In line with its role in the FA pathway, defects in FANCJ give rise to exquisite ICL sensitivity in a range of different species (Bridge et al. 2005; Levitus et al. 2005; Litman et al. 2005; Youds et al. 2008). However, attempts to place FANCJ within the FA pathway have provided little insight into its precise function in ICL repair. FANCJ is dispensable for the activation of the FA core complex and hence the monoubiquitylation of FANCD2/FANCI and its recruitment to ICL lesions (Levitus et al. 2004; Litman et al. 2005). Furthermore, an interaction between FANCJ and BRCA1 is not required for classical ICL repair (Xie et al. 2010b), and a role for FANCJ in HR downstream from ICL incision remains ambiguous. Currently, the function of FANCJ in ICL repair remains poorly defined.

Biochemical studies have shown that FANCJ unwinds a variety of DNA substrates, including 5′ flaps, forked duplexes, D loops, 5′ tailed triplexes, and G4-DNA structures in a 5′–3′ direction in vitro (Gupta et al. 2005; London et al. 2008; Wu et al. 2008; Sommers et al. 2009). Of these DNA structures, a clear picture has emerged linking FANCJ to the metabolism of G4-DNA secondary structures in vivo. Such a role was first suggested from the observation of increased G/C tract deletions in *dog-1* (deletion of G tracts; *Caenorhabditis elegans* FANCJ) mutant worms, which was proposed to reflect a defect in unwinding G4-DNA structures formed within G/C tracts (Cheung et al. 2002; Youds et al. 2008). Subsequent studies in human *Fancj*-deficient cells found that genomic deletions tend to accumulate in the vicinity of potential G4-DNA-forming sequences (London et al. 2008). Genome-wide transcription profiling of FANCJ knockout chicken DT40 cells has also revealed that dysregulated genes are significantly associated with G4 sequences. It was proposed that FANCJ maintains epigenetic stability near G4-DNA motifs through two independent mechanisms dependent on either the Y family polymerase REV1 or WRN/BLM helicases (Sarkies et al. 2012). Recently, it was reported that FANCJ depletion from *Xenopus laevis* egg extract leads to persistent replication stalling at G4 sequences (Castillo Bosch et al. 2014). Despite these observations, it is currently unclear whether FANCJ functions exclusively to maintain genome stability associated with G4-DNA-forming sequences or also participates in the metabolism of other DNA secondary structures.

In this study, we report that *Fancj*-null mice exhibit subfertility, germ cell attrition, epithelial tumor predisposition, and exquisite sensitivity to ICL-inducing agents, which phenocopy other mouse models of FA (Bakker et al. 2012). Unexpectedly, *Fancj*-deficient mice also present with enhanced predisposition to lymphoma, and cells derived from these mice are hypersensitive to replication inhibitors. Furthermore, *Fancj*^−/−^*Fancd2*^−/−^ double-knockout mice display heightened germ cell attrition and are considerably more sensitive to ICL-inducing agents than single knockouts. Since *Fancj*-deficient cells are insensitive to G4-stabilizing drugs and are devoid of telomere fragility, we considered the possibility that FANCJ performs additional functions in genome stability independent of its role in the FA pathway and distinct from a role in G4-DNA metabolism. Strikingly, we show that *Fancj*-deficient, but not *Fancd2*-deficient, mice accumulate spontaneous MSI corresponding to both expansions and contractions of repeat sequences. Similarly, FA-J patient cells and human *Fancj* knockouts cells also present with MSI, which is exacerbated by replication inhibition. Thus, we propose that FANCJ counteracts the formation of secondary structures that arise during replication of microsatellite sequences, which minimizes the potential for strand slippage during DNA polymerization. Our findings can potentially explain the widespread involvement of FANCJ in human cancers.

## Results

### Generation of *Fancj*-deficient mice

**Figure 1.**
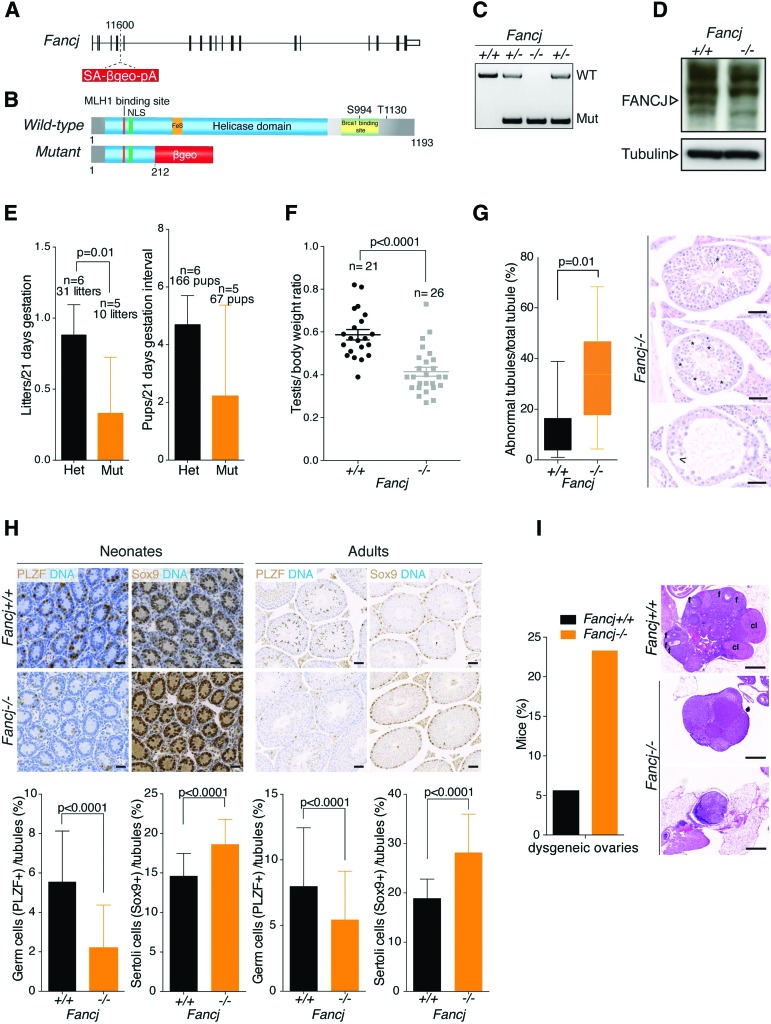
Germ cell attrition and subfertility in *Fancj* knockout mice. (*A*) Schematic representation of the mouse *Fancj* genomic locus. The pGT0Lxf gene trap vector is inserted at position 11600. The whole genomic locus is 143.06 kb, and introns (lines) and exons (bars) are represented to scale. (*B*) FANCJ protein organization. Note that the *Fancj* mutant protein is truncated at 212 amino acids and fused with the β-geo cassette of the gene trap vector. (*C*) *Fancj* PCR genotyping strategy. (*D*) Western blot on *Fancj*^+/+^ and *Fancj*^−/−^ MEF lysates. The arrow indicates the FANCJ protein. Note that FANCJ is not expressed in *Fancj*^−/−^ cells. Tubulin was used as loading control. (*E*) Fertility comparison between *Fancj* heterozygous and mutant breedings. Each pair bred during 5–6 mo, and the number of litters and pups per litter was quantified per 21-d gestation. Significance: *t*-test, litter per 21 d gestation, *P* = 0.01; pups per 21 d gestation, *P* = 0.08. (*F*) *Fancj* testis weights. Each testis weight was normalized against mice body weight. Significance: *t*-test, *P* < 0.0001. (*G*, *left*) Abnormal seminiferous tubule quantification on *Fancj* testis sections. (*Right*) Representative *Fancj*^−/−^ seminiferous tubule images are shown. The *Fancj*^−/−^ testis contains both normal tubules and also different levels of atrophic tubules. The *bottom* picture shows a severely atrophic tubule with almost no spermatogenic cells remaining. Asterisks show tubule degeneration. Bars, 50 µm. Significance: *t*-test, *P* = 0.01. (*H*, *top*) Five-day-old neonate and adult seminiferous tubules stained with PLZF (germ cells; brown) and Sox9 (sertoli; brown) markers. DNA is counterstained with hematoxylin (blue). Bars, 50 µm. (*Bottom*) Spermatogonia and sertoli cell quantification per tubule. Error bars represent ±standard error of the mean (SEM) of at least 50 tubules. Significance: with *t*-test, *P* < 0.0001. (*I*, *left*) Dysgeneic ovary frequency. *n* = 43 *Fancj*^−/−^; *n* = 18 *Fancj*^+/+^. (*Right*) Representative images of dysgeneic ovaries. Bars, 500 µm. (f) Follicles; (cl) corpus luteum. Significance: Fisher's exact test, *P* = 0.1.

To investigate the function of FANCJ in vivo, we generated a *Fancj* mutant mouse from an existing gene trap embryonic stem cell line (RRI409). Insertion site mapping by splinkerette PCR revealed the gene trap cassette to be integrated into the fifth intron of the *Fancj* mouse gene, which is predicted to truncate the gene within the critical helicase motifs (Fig. 1A,B; Supplemental Fig. S1A; Devon et al. 1995). This facilitated development of a genotyping strategy, which was used to identify wild-type, heterozygous, and mutant mouse embryonic fibroblasts (MEFs) derived from heterozygous mice (Fig. 1C). Quantitative RT–PCR analysis failed to detect Fancj mRNA expression (exon 5–6 junction) or expression of a fusion transcript between exon 5 and the β-geo cassette (Supplemental Fig. S1B). Western blotting of whole-cell extracts with an antibody raised against the conserved N terminus of FANCJ protein also confirmed the absence of a truncated or fusion protein (∼170 kDa) (Fig. 1D; Supplemental Fig. S5A). These data suggest that the gene trap cassette eliminates expression of the FANCJ ORF, resulting in a null allele.

### *Fancj*-deficient mice are subfertile and present with germ cell attrition

Characterization of *Fancj*^−/−^ mice indicated that they are viable and born at expected Mendelian ratios (Supplemental Fig. S1C), as has also been reported for other FA and FA-like knockout mice, including *Fancd2-* and *Helq*-deficient mice (Houghtaling et al. 2003; Adelman et al. 2013). *Fancj*^−/−^ mice displayed normal growth and weight gain when compared with wild-type littermates up to the age of 19 wk and were devoid of any noticeable developmental abnormalities (Supplemental Fig. S1D). However, comparison of mating ability between six *Fancj*^+/−^ heterozygous and five *Fancj*^−/−^ homozygous pairs mated continuously over a 5-mo period revealed reduced litter numbers and size, consistent with subfertility. Indeed, *Fancj*^+/−^ heterozygous matings resulted in 31 litters with 166 pups (0.9 litter per 21-d gestation interval and 4.7 pups per litter), whereas *Fancj*^−/−^ homozygous matings produced only 10 litters and 66 pups (0.3 litter per 21-d gestation interval and 2.2 pups per litter) (Fig. 1E). This observation raised the possibility that, like other FA mouse models, FANCJ deficiency is also associated with subfertility.

To identify the possible cause of this fertility defect, we first examined testes from *Fancj*^−/−^ mutant and wild-type littermates. Similar to other FA mice, *Fancj*^−/−^ mice have reduced testis size and weight relative to wild-type controls (Fig. 1F), which are not significantly altered by age (Supplemental Fig. S1E). Histological analysis of *Fancj*^−/−^ adult testes revealed many normal tubules populated with the full complement of spermatogonia; however, regions of atrophy were also evident. Thirty-four percent of seminiferous tubules were found to be atrophic, compared with 11% in controls (Fig. 1G). The source of subfertility of *Fancj*^−/−^ mice was not limited to the male gonad, as 23% of female gonads analyzed at 35 wk exhibited ovarian dysgenesis, compared with 5.6% in controls. Histological analysis confirmed that only a few primary or no developing follicles were present in *Fancj*^−/−^ ovaries (Fig. 1I).

Closer scrutiny of adult *Fancj*^−/−^ testis sections revealed that the levels of atrophy in seminiferous tubules were stochastic, ranging from mild to severe atrophy, with some tubules devoid of all spermatogenic layers (Fig. 1G). Since further analysis of the male gonad failed to reveal a specific spermatogenesis defect in *Fancj*^−/−^ mice, it was most likely that atrophy arose from a stem cell problem, which we sought to assess by staining adult testis sections with the spermatogonial stem cell marker PLZF. A marked reduction in the number of PLZF-positive cells per tubule was observed in the *Fancj*^−/−^ mutant testes compared with controls (5.5 ± 0.4 vs. 8.0 ± 0.5, respectively). Correspondingly, the number of sertoli cells stained by the Sox9 sertoli-specific marker was significantly increased in the *Fancj*^−/−^ mutant mice relative to controls (28 ± 0.8 vs. 18 ± 0.4, respectively) (Fig. 1H, right). As the levels of testis atrophy in the *Fancj*^−/−^ mutant mice did not noticeably worsen with age, we considered that the germ cell defect likely arises during development. To assess this possibility, we examined spermatogonia numbers in 5-d-old neonate testes. *Fancj*^−/−^ mutant testes presented with a 2.5-fold decrease in the number of PLZF-positive spermatogonia when compared with control testes. Similarly, we also observed a corresponding increase in the number of sertoli cells in the mutant testes (Fig. 1H, left). These observations suggest that loss of FANCJ leads to subfertility as a result of germ cell attrition during development.

### *Fancj*^−/−^ MEFs are exquisitely sensitive to ICLs

**Figure 2.**
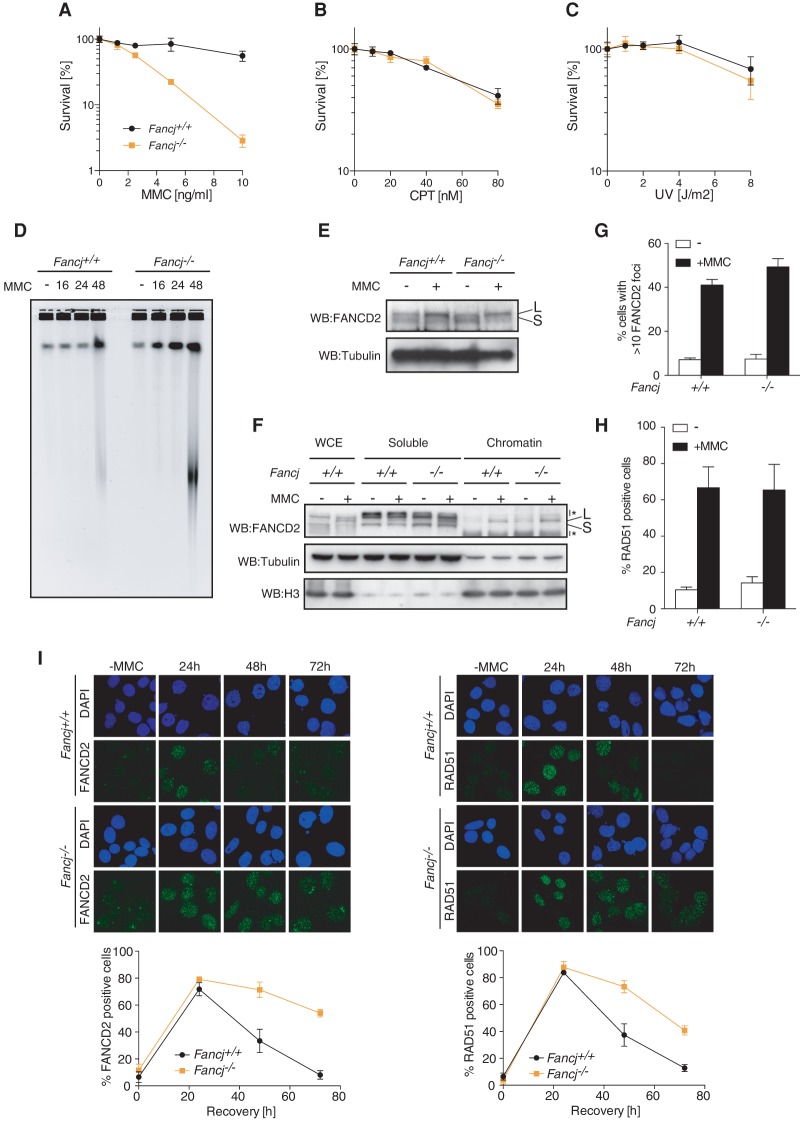
DNA damage sensitivity and tumor predisposition in *Fancj* knockout mice. (*A*–*C*) Clonogenic survival assays of *Fancj*^+/+^ and *Fancj*^−/−^ MEFs exposed to the indicated DNA damage. The error bar represents SEM. (*D*) Pulse field gel electrophoresis of samples harvested at the indicated time points after release from 1 µg/mL MMC treatment. (*E*) FANCD2 Western blot of whole-cell lysates from *Fancj*^+/+^ and *Fancj*^−/−^ MEFs in the absence and presence of MMC. (*F*) Chromatin fractions from *Fancj*^+/+^ and *Fancj*^−/−^ MEFs with or without MMC treatment were probed for FANCD2, tubulin, and histone H3 antibodies. Asterisks indicate nonspecific bands. (*G*) Quantification of FANCD2-positive cells in *Fancj*^+/+^ and *Fancj*^−/−^ MEFs with or without MMC treatment. The error bar indicates SD. (*H*) Quantification of RAD51-positive cells in *Fancj*^+/+^ and *Fancj*^−/−^ MEFs after MMC treatment. Error bar indicates SD. (*I*, *left*) Representative images of FANCD2 foci (*top*) and quantification (*bottom*) at the indicated time points after release from MMC treatment. (*Right*) Representative images of RAD51 foci (*top*) and quantification (*bottom*). The error bar indicates SD.

Since subfertility and germ cell attrition are common features of FA, we next assessed whether *Fancj*^−/−^ mice exhibit other FA phenotypes, including cellular sensitivity to ICL agents. *Fancj*^−/−^ MEFs exhibited exquisite sensitivity to the ICL-inducing agent mitomycin C (MMC) but not camptothecin or UV (Fig. 2A–C) and accumulated radial chromosomes, a hallmark of FA (Supplemental Fig. S5B). Analysis of ICL incision at MMC lesions was assessed by monitoring the accumulation of DSB intermediates by pulse field gels following treatment of cells with MMC. Heightened levels of DSBs accumulated in *Fancj*^−/−^ MEFs with accelerated kinetics when compared with wild-type controls (Fig. 2D). These results suggest that MMC-induced lesions are incised, but the damage persists in *Fancj*^−/−^ MEFs, indicating that lesion repair is compromised downstream from ICL incision.

To further examine the nature of the ICL repair defect in *Fancj*-deficient cells, we monitored the integrity of two key events in ICL repair; namely, the monoubiquitylation of FANCD2 by the FA core complex and its subsequent assembly into chromatin-associated repair foci. Monoubiquitylated FANCD2 was induced to similar levels in both wild-type and *Fancj*^−/−^ MEFs in response to MMC treatment (Fig. 2E). Furthermore, FANCD2 was readily detected in the chromatin fraction and in repair foci in both wild-type and *Fancj*^−/−^ MEFs following MMC (Fig. 2F,G). In the canonical ICL repair pathway, DSBs are processed by Rad51-mediated HR. To assess the effect of FANCJ on early steps of HR, we monitored Rad51 and FANCD2 focus formation. Similar levels of RAD51 foci were observed in *Fancj*^+/+^ and *Fancj*^−/−^ MEFs after MMC (Fig. 2H). However, in *Fancj*^−/−^ MEFs, FANCD2 and RAD51 foci persist much longer than in *Fancj*^+/+^ MEFs (Fig. 2I), suggesting that FANCJ is required for ICL repair and is important for the timely resolution of FANCD2- and RAD51-marked repair intermediates.

### *Fancj*^−/−^ mice are tumor-prone and predisposed to epithelial cancers

**Figure 3.**
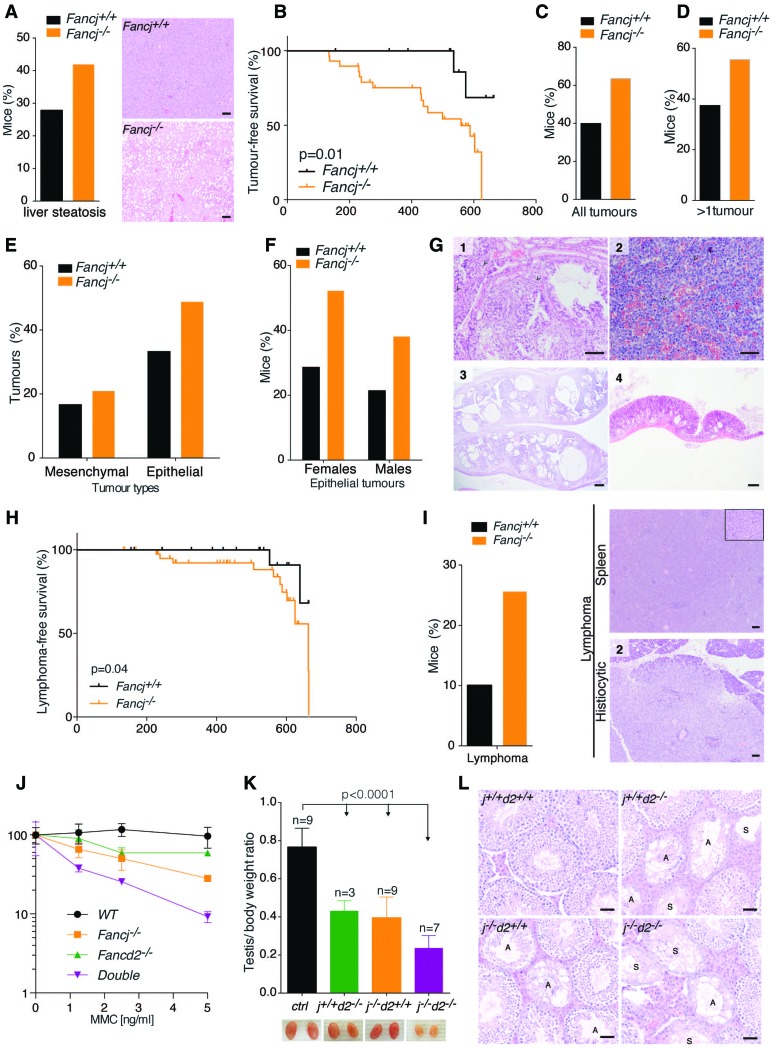
*Fancj* knockout mice exhibit phenotypes distinct from the canonical FA phenotype. (*A*, *left*) Liver steatosis frequency in *Fancj* mice. (*Right*) Representative images. Bars, 100 µm. Significance: Fisher's exact test, *P* = 0.1. (*B*) Tumor-free survival of *Fancj* mice. Significance: Mantel-Cox test, *P* = 0.01. *n* = 28 *Fancj*^−/−^; *n* = 12 *Fancj*^+/+^. Mice culled due to nonspecific phenotypes (e.g., dermatitis, overgrown teeth, and fits) were excluded from this study. (*C*) Frequency of mice with tumors. Significance: Fisher's exact test, *P* = 0.05. (*D*) Frequency of mice with more than one tumor. Significance: Fisher's exact test, *P* = 0.08. (*E*) Frequency of mesenchymal and epithelial tumors in *Fancj* mice. Significance: Fisher's exact test, mesenchymal tumors, *P* = 0.5; epithelial tumors, *P* = 0.1. (*F*) Frequency of epithelial tumors in *Fancj* males and females. Significance: Fisher's exact test, females, *P* = 0.1; males, *P* = 0.15. (*G*) Representative images of epithelial tumors. (Panel *1*) Harderian gland adenoma with some compressed normal acini at the top of the field. The adenomatous cells have large, darker nuclei with some mitotic figures (arrows). Bar, 100 µm. (Panel *2*) A high-magnification view of a pituitary adenoma with erythrocyte-filled vascular channels and a central mitosis (arrows). Bar, 50 µm. (Panel *3*) Both uterine horns are enlarged due to cystic endometrial hyperplasia with variable glands, some of which are cystically dilated. Bar, 500 µm. (Panel *4*) A low-magnification view of a colonic adenoma with low-grade dysplasia situated above the muscularis propria, with a smaller, apparently separate, adenomatous area at the right. Bar, 500 µm. (*H*) Lymphoma-free survival of *Fancj* mice. Significance: Mantel-Cox test, *P* = 0.04. *n* = 43 *Fancj*^−/−^; *n* = 20 *Fancj*^+/+^. (*I*, *left*) Frequency of lymphomas in *Fancj* mice. Significance: Fisher's exact test, *P* = 0.1. (*Right*) Representative images of lymphomas. Bars, 50 µm. (*J*) Clonogenic survival assays of the indicated cell lines exposed to MMC. The error bar represents SEM. (*K*) *Fancj/Fancd2* testis weights. Each testis weight was normalized against mouse body weight. Significance: one-way ANOVA, *P* < 0.0001. (*L*) Representative images of *Fancj/Fancd2* seminiferous tubule atrophy. (A) Atrophic tubules; (S) sertoli cells tubules. Bars, 50 µm.

To study the impact of FANCJ deletion in aging mice, we monitored a cohort of 21 *Fancj*^+/+^ and 44 *Fancj*^−/−^ mice over the course of their lifetime. Aged *Fancj*^−/−^ mutant mice presented with lipid accumulation in the liver (Fig. 3A), and their tumor-free survival was found to be significantly reduced compared with control mice; 50% of the mutant mice presented with tumors by 559 d (Fig. 3B). Seventy-four percent of *Fancj*^−/−^ mutants developed tumors, with a greater incidence in females (∼74% vs. ∼48% only in males) (Fig. 3C; Supplemental Fig. S2A), and 60% of the *Fancj*^−/−^ mutant mice presented with more than one primary tumor (Fig. 3D). Similar to other FA mouse models, one of the most common tumors in *Fancj*^−/−^ mice is of epithelial origin (Houghtaling et al. 2003). Indeed, 21 epithelial tumors developed in 44 *Fancj*^−/−^ mutant animals, which represent an incidence of 49% (Fig. 3E; Supplemental Fig. S2B). Mutant females presented with more epithelial tumors when compared with males in the cohort, which corresponds to a frequency of ∼52% in females and 39% in males (Fig. 3F). Mutant females were particularly prone to cystic ovaries (four tumors out of 23 animals), endometrial hyperplasia (three tumors out of 23 animals), and pituitary gland adenomas (three tumors out of 23 animals), whereas Harderian gland adenomas were the most prominent epithelial tumors in males (five tumors out of 21 animals) (Fig. 3G; Supplemental Fig. S2C). Thus, loss of FANCJ leads to an increased predisposition to epithelial tumors in both males and females, as has been shown in other FA mouse models.

### *Fancj*^−/−^ mice exhibit predisposition to lymphomas

Despite the tumor spectrum of *Fancj*^−/−^ mice being similar to that of other mouse models of FA, we also observed that epithelial tumors are not the only prominent tumor type in *Fancj*^−/−^ mice. Surprisingly, the lymphoma-free median survival age, although late in life, was significantly decreased in *Fancj*^−/−^ mice (Fig. 3H), with 26% of *Fancj*^−/−^ mutants developing lymphomas compared with ∼10% in littermate controls (Fig. 3I). This was in contrast to *Fancd2*^−/−^ and *HelQ*-deficient FA mouse models, which do not present with heighted lymphoma predisposition (Supplemental Fig. S3A,B; Houghtaling et al. 2003). Further interrogation of the *Fancj*^−/−^ lymphoma-free median survival data revealed that mutant females are more susceptible than males to lymphomas, with an incidence of ∼40% compared with 9.5% in mutant males (Supplemental Fig. S3C). Histological analysis revealed that lymphomas were widely spread in most animals, with an increased frequency in the spleen, B cells, and mesenteric and salivary lymph nodes (Supplemental Fig. S3D). This unexpected difference in the tumor spectrum of *Fancj*-deficient mice compared with other FA mouse models raised the possibility that FANCJ performs roles that are independent of the canonical FA pathway, at least with respect to preventing lymphoma development.

### Loss of *Fancd2* exacerbates the *Fancj* mutant phenotype

Given the sensitivity to MMC, germ cell attrition, and predisposition to epithelial tumors associated with FANCJ deficiency overlap with the phenotype of other mouse models of FA, yet lymphoma predisposition is unique to FANCJ, we were compelled to examine whether FANCJ actually functions genetically in the FA pathway. To this end, we crossed *Fancj*^−/−^ and *Fancd2*^−/−^ mutant mice and subjected the resulting *Fancj^−/−^Fancd2*^−/−^ double-mutant mice to genetic epistasis analysis. Unexpectedly, MEFs derived from *Fancj^−/−^Fancd2*^−/−^ double-mutant mice were significantly more sensitive to MMC than either single mutant (Fig. 3J). The increased sensitivity in the double mutant implies that FANCJ may function in parallel to or performs roles that are distinct from the canonical FA pathway in ICL repair.

We also examined the genetic relationship between FANCJ and FANCD2 in mice. *Fancj^−/−^Fancd2*^−/−^ double-mutant mice appeared grossly normal and healthy, similar to their single-mutant counterparts, and were born at near to expected Mendelian ratios (χ^2^, 9.8) (Supplemental Fig. S3E). However, when testes were examined, double mutants possessed significantly smaller testes than the controls. There is also a subtle additive but not significant decrease of testis size between *Fancj*^−/−^ or *Fancd2*^−/−^ and *Fancj^−/−^Fancd2*^−/−^ (Fig. 3K). Histology analysis revealed the presence of significantly more atrophic tubules in the double mutant when compared with single mutants and controls, which were also mostly devoid of spermatogenic cells and composed of sertoli cells only (Fig. 3L; Supplemental Fig. S3F). Together with the increased MMC sensitivity and lymphoma predisposition, these data suggest that FANCJ performs cellular and organismal roles that are distinct and independent of the FA pathway.

### *Fancj*^−/−^ MEFs exhibit spontaneous DNA damage and aphidicolin sensitivity

**Figure 4.**
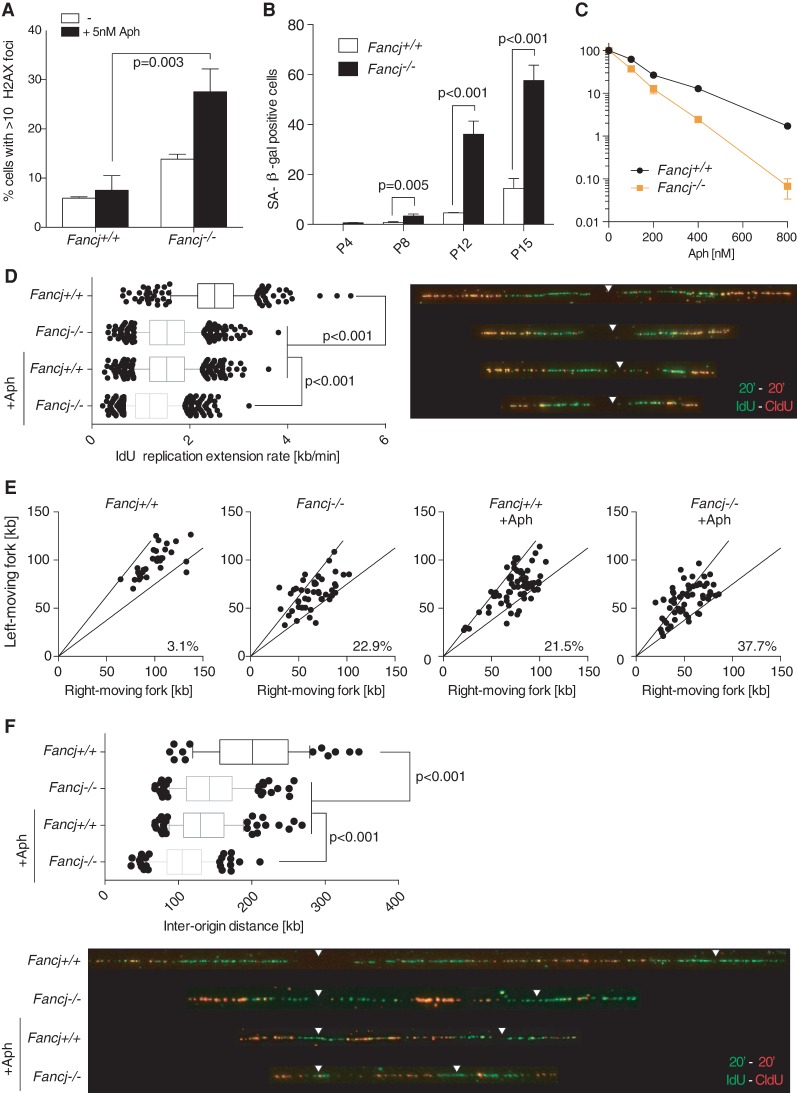
Spontaneous DNA damage, senescence, and replication stress in *Fancj* knockout cells. (*A*) Quantification of γH2AX-positive cells in *Fancj*^+/+^ and *Fancj*^−/−^ MEFs in the absence or presence of 5 nM aphidicolin (Aph). The error bar indicates SD. (*B*) Quantification of SA-β-gal-positive cells in *Fancj*^+/+^ and *Fancj*^−/−^ primary MEFs at the indicated passage numbers. The error bar indicates SD. (*C*) Clonogenic survival assays of *Fancj*^+/+^ and *Fancj*^−/−^ MEFs exposed to aphidicolin. The error bar represents SEM. (*D*) Replication rates in *Fancj*^+/+^ and *Fancj*^−/−^ MEFs in the absence or presence of aphidicolin. Nascent DNA strands were pulse-labeled with iodo-deoxyuridine (IdU) and chloro-deoxyuridine (CldU) for 20 min, individually. More than 300 fibers in the indicated cell lines were measured. Arrowheads indicate the potential position of replication origin. The error bar indicates SD. (*E*) Quantification of asymmetry between sister replication forks in the indicated MEFs with or without aphidicolin. The areas between the black lines contain sister forks with <25% length difference. (*F*) Interorigin distances in *Fancj*^+/+^ and *Fancj*^−/−^ MEFs with or without aphidicolin. The error bar shows SD.

To gain further insight into the functions of FANCJ in maintaining genome integrity, we examined *Fancj*^−/−^ MEFs for markers of spontaneous DNA damage. In contrast to wild-type controls, *Fancj*^−/−^ MEFs exhibit spontaneous γH2AX foci even in the absence of DNA-damaging agents (Fig. 4A). To examine whether the elevated levels of DNA damage influence cell growth, cellular senescence was monitored in primary cells (Fig. 4B). Primary *Fancj*^−/−^ MEFs were found to undergo spontaneous senescence at later passages, which was not observed in wild-type controls. These observations suggest that *Fancj*^−/−^ MEFs undergo senescence due to increased levels of spontaneous DNA damage. Consistent with the DNA damage arising during DNA replication, we observed that *Fancj*^−/−^ MEFs exhibit hypersensitivity to low doses of the replication inhibitor aphidicolin (Fig. 4C). In addition, the levels of γH2AX foci were significantly increased in *Fancj*^−/−^ MEFs after aphidicolin treatment (Fig. 4A). These results support that FANCJ also has a noncanonical FA function.

### FANCJ is required for normal replication fork progression

The spontaneous DNA damage and aphidicolin sensitivity of *Fancj*^−/−^ MEFs raised the possibility that FANCJ is required to facilitate normal DNA replication. To examine this possibility, we assessed replication fork dynamics by monitoring iodo-deoxyuridine and chloro-deoxyuridine (IdU/CldU) incorporation by DNA combing. As shown in Figure 4D, loss of FANCJ results in significantly slower replication fork extension rates in unchallenged cells relative to control cells (1.56 and 2.51 kb/min, respectively), and this is further exacerbated by aphidicolin treatment (1.24 kb/min). By measuring the length of the left and right moving fork, we also observed elevated levels of asymmetric replication forks in *Fancj*^−/−^ MEFs compared with wild-type controls (22.9% vs. 3.1%) under both unchallenged and aphidicolin-treated conditions (Fig. 4E). These data suggest that loss of FANCJ results in increased replication fork stalling and/or collapse. Consistent with the reduced fork extension rates, interorigin distances under both unchallenged and aphidicolin-treated conditions were significantly shorter in *Fancj*^−/−^ MEFs relative to wild-type controls (145.3 and 203.7 kb in untreated, and 107.7 and 136.3 kb in treated, respectively). These results establish that FANCJ facilitates normal DNA replication and plays a crucial role in DNA replication when replication forks are perturbed.

### *Fancj*^−/−^ MEFs are not sensitive to G4-stabilizing drugs

**Figure 5.**
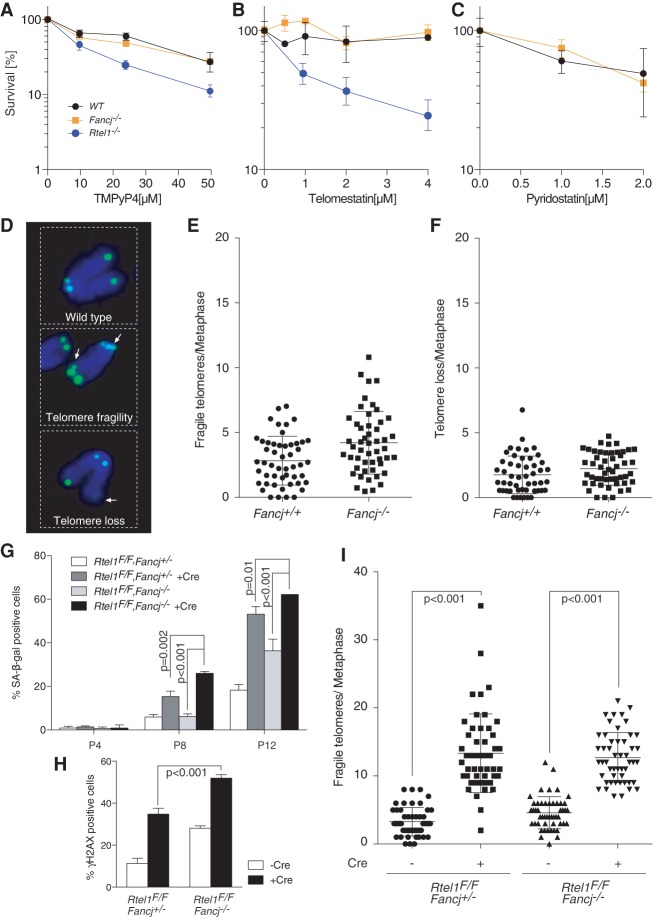
*Fancj* knockout cells are not sensitive to G4-DNA-stabilizing drugs and do not present with telomere fragility. (*A*–*C*) Clonogenic survival assays of *Fancj*^+/+^, *Fancj*^−/−^, and *Rtel1*^−/−^ MEFs exposed to TMPyP4 (*A*), telomestatin (*B*), and pyridostatin (*C*). The error bar represents SEM. (*D*) Representative image of fragile telomeres and telomere loss. (*E*,*F*) Quantification of fragile telomeres (*E*) and telomere loss (*F*) per metaphase in *Fancj*^+/+^ and *Fancj*^−/−^ MEFs. The error bar indicates SD. Significance: *t*-test, *P* = 0.08 in *E*; *P* = 0.3 in *F*. (*G*) Quantification of SA-β-gal-positive cells in the indicated primary MEFs with or without Cre treatment at passages 4, 8, and 12. The error bar indicates SD. (*H*) Quantification of γH2AX-positive cells in the indicated MEFs with or without Cre treatment. The error bar indicates SD. (*I*) Quantification of fragile telomeres per metaphase in the indicated MEFs with or without Cre treatment. The error bar represents SD. Significance between *Rtel1^f/f^ Fancj*^+/−^ + Cre and *Rtel1^F/F^ Fancj*^−/−^ + Cre: *t*-test, *P* = 0.5.

Biochemical studies have shown that FANCJ is proficient at unwinding G4-DNA structures in vitro (London et al. 2008) and can promote DNA replication through G4 structures in a *X. laevis* cell-free system (Peng et al. 2014). A role for FANCJ in unwinding G4-DNA structures in vivo is also suggested by the accumulation of large genomic deletions in the vicinity of G-rich sequences in *dog-1* (*C. elegans* FANCJ) mutant worms (Cheung et al. 2002) and human cells lacking FANCJ (London et al. 2008). These observations prompted us to test whether the replication problems detected in *Fancj*-deficient cells reflect a function in counteracting G4-DNA structures. To this end, we first analyzed the sensitivities of *Fancj*^−/−^ MEFs to a range of G4-stabilizing agents. Unexpectedly, *Fancj*^−/−^ MEFs exhibit comparable sensitivity to TMPyP4, telomestatin, and pyridostatin compared with wild-type controls. In contrast, *Rtel1*-deficient MEFs exhibit sensitivity to TMPyP4 and telomestatin, as previously reported (Fig. 5A–C).

### Telomeric G4-DNA sequences are stable in *Fancj*^−/−^ MEFs

Next, we examined a possible role for FANCJ in unwinding G4-DNA sequences that form within the TTAGGG repeats at vertebrate telomeres. Defects in counteracting telomeric G4-DNA structures during telomere replication give rise to telomere fragility, which is observed as multiple spatially distinct telomere FISH signals at chromosomes ends (Sfeir et al. 2009). Consistent with the lack of sensitivity to G4-stabilizing agents, the levels of telomere fragility and telomere loss in *Fancj*^−/−^ MEFs were indistinguishable from wild-type controls (Fig. 5E,F). This is in contrast to *Rtel1*-deficient MEFs, which exhibit extensive telomere fragility (Vannier et al. 2012). Prompted by our previous observation that the *rtel-1 dog-1* (*C. elegans* RTEL1 and FANCJ) double mutant is synthetic-lethal due to catastrophic genome instability (Barber et al. 2008), we generated conditional *Rtel1^F/F^ Fancj*^−/−^ double-mutant MEFs to investigate a potential redundant role for FANCJ in unwinding G4-DNA structures in the absence of RTEL1. Consistent with our previous findings in worms, *Rtel1^−/−^Fancj*^−/−^ double-knockout MEFs display accelerated senescence (Fig. 5G) and elevated levels of spontaneous γH2AX foci relative to single-knockout MEFs (Fig. 5H). However, the levels of telomere fragility observed in *Rtel1^F/F^Fancj*^−/−^ double-knockout MEFs was not significantly different from the levels present in the *Rtel1*^−/−^ single-knockout cells (Fig. 5I)*.* These data suggest that mouse FANCJ is dispensable for the maintenance of telomeric G4 sequences and potentially other G4-DNA sequences.

### MSI in *Fancj*^−/−^ MEFs

**Figure 6.**
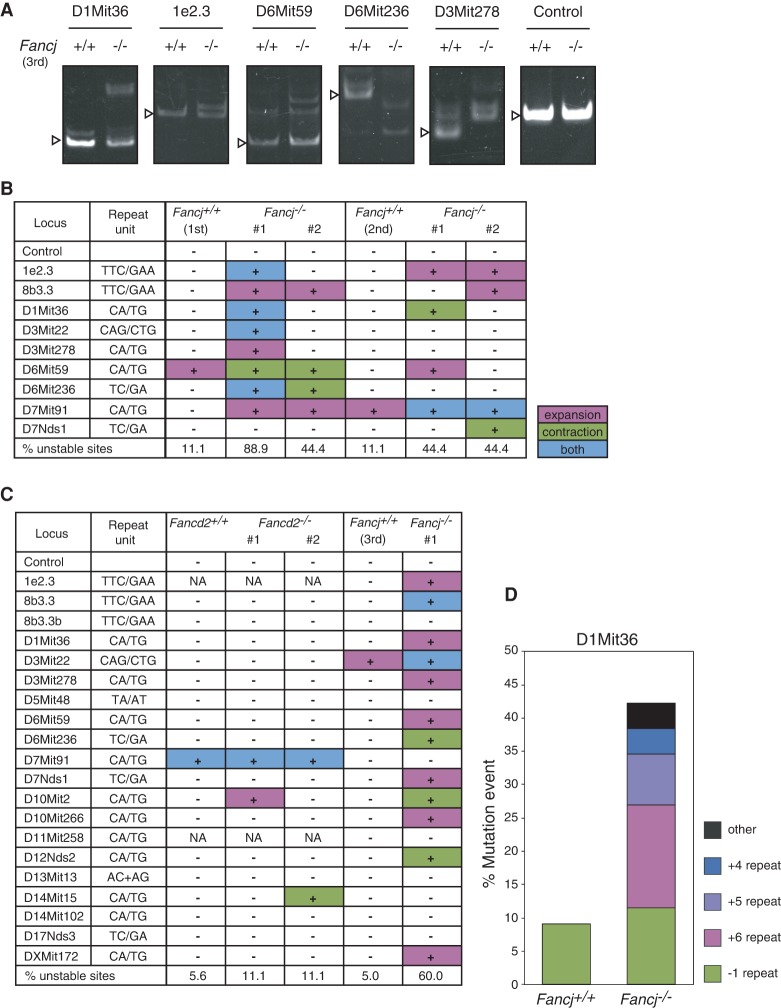
*Fancj* knockout mice exhibit MSI independent of the canonical FA pathway. (*A*) Representative images of MSI at the indicated loci in MEFs. (*B*) Microsatellite analysis in two independent sets of *Fancj*^+/+^ and *Fancj*^−/−^ cell lines. MSI status is indicated as unstable (+) and stable (−). Types of instability are indicated as expansion (magenta), contraction (green), and both (blue). (*C*) MSI in *Fancd2*^−/−^ and *Fancj*^−/−^ cell lines. MSI status and types of instability are indicated as in *B*. (*D*) Distribution of mutation types at the D1Mit36 locus. D1Mit36 PCR products were cloned into empty vector and sequenced. The percentage of mutation type is calculated. DNA sequences are shown in Supplemental Figure S4A.

In the absence of any overt phenotype associated with G4-DNA metabolism in *Fancj-*deficient cells, we considered the possibility that problems associated with other types of DNA sequences/secondary structures are the cause of the replication defects in *Fancj*^−/−^ MEFs. Previous studies reported that FANCJ physically interacts with the MMR protein MLH1 (Cannavo et al. 2007; Peng et al. 2007), but the functional relevance of this association remains unknown. Intriguingly, MMR deficiency is associated with MSI, which reflects a failure to correct base–base mismatches and short deletions that arise following template slippage at these short tandem repeated sequences. Since MMR-deficient mice are also associated with increased risk of lymphoma (Li 2008), we considered the possibility that the replication defects in *Fancj*^−/−^ cells might arise from problems at microsatellite sequences. To test this hypothesis, we first derived wild-type and two *Fancj*^−/−^ (#1 and #2) primary MEF lines from timed matings of *Fancj*^+/−^ heterozygous mice and examined the integrity of nine distinct microsatellites, which were chosen based on their reported instability in MMR-deficient cells. PCR was used to assess MSI and was defined as a deviation from the wild-type banding pattern, with higher molecular bands and lower molecular bands classified as microsatellite expansion and microsatellite contraction, respectively. Strikingly, both *Fancj*^−/−^ MEF lines (#1 and #2) exhibited instability associated with eight out of nine (#1) and four out of nine (#2) microsatellites, which included both expansion and contraction (Fig. 6B). To confirm this result, we independently derived a wild-type and two further *Fancj*^−/−^ MEF lines from a second timed mating of *Fancj*^+/−^ heterozygous mice. Unlike the wild-type control cells, both *Fancj*^−/−^ MEF lines exhibited instability associated with four out of nine (#1) and four out of nine (#2) microsatellites (Fig. 6B).

To investigate whether MSI is also observed in other mouse models of FA, we analyzed the integrity of 18 microsatellite sequences in two *Fancd2*^−/−^ primary MEF lines (#1 and #2) and compared these with wild-type and *Fancj*^−/−^ primary MEF lines derived from a third independent mating. FANCD2 was analyzed, as it is a key component of the FA pathway and is regulated by the FA core complex. In contrast to the MSI evident in *Fancj*^−/−^ primary cells (12 of 20), the two *Fancd2*^−/−^ primary MEFs (1: two of 18; #2: two of 18) were largely devoid of unstable microsatellite sequences, similar to wild-type cells (one of 18; one of 20) (Fig. 6C). These data suggest that MSI is likely unique to FANCJ-deficient cells among the defined FA genes.

To further analyze the sequence changes associated with MSI in the absence of FANCJ, we cloned and sequenced the microsatellite D1Mit36 from wild-type and *Fancj*^−/−^ MEFs (Fig. 6D; Supplemental Fig. S4A). Sequencing of the PCR products from the wild type showed only two sequences, which likely correspond to the microsatellites on each homologous chromosome. In contrast, six different sequence changes were detectable from *Fancj*^−/−^ cells, which corresponded to a single-repeat contraction, four- to six-repeat expansions, and more complex alterations. These observations are consistent with increased template slippage at microsatellites, which could occur if extruded secondary structures formed from the tandem repeats persist in the absence of FANCJ.

### Human FA-J patient cells and CRISPR-derived *Fancj*^−/−^ U2OS cells exhibit MSI, which is exacerbated by aphidicolin treatment

**Figure 7.**
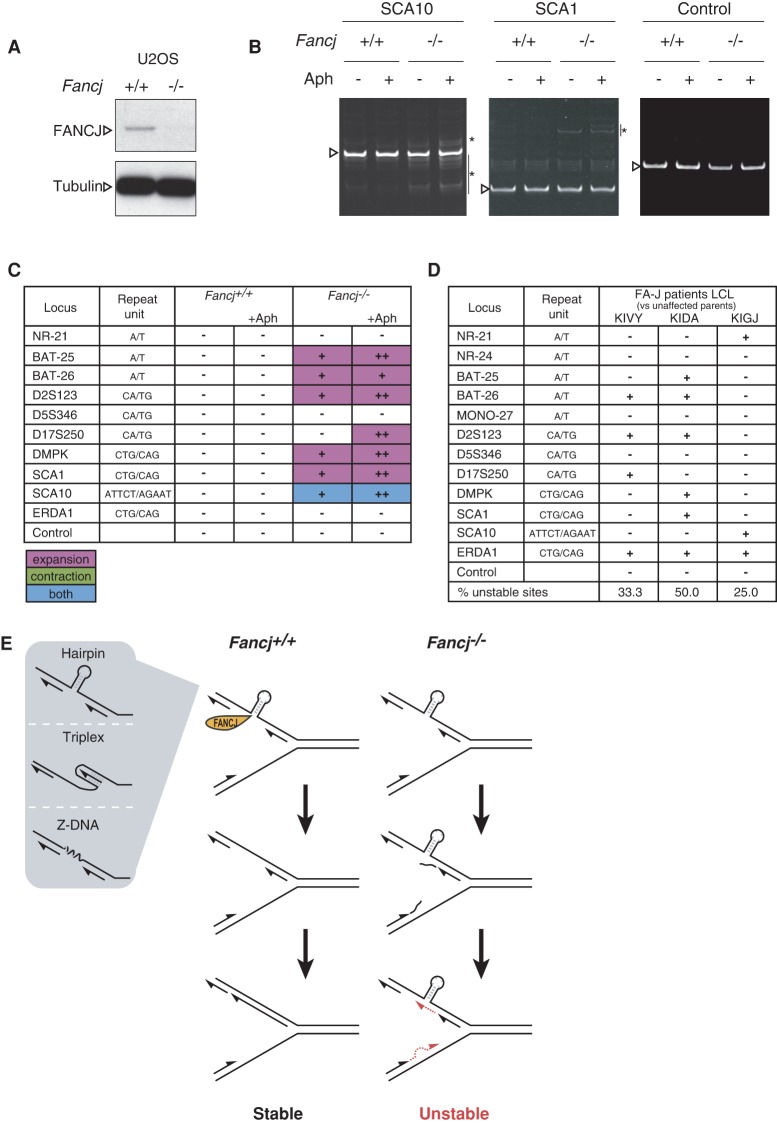
MSI in human FANCJ-deficient patient and knockout cells. (*A*) Western blot with FANCJ antibody confirmed the loss of detectable FANCJ protein in control (+/+) and CRISPR knockout (−/−) U2OS cells. (*B*) Representative images of MSI at the indicated loci in U2OS cells. Asterisks indicate PCR products specifically found in *Fancj* knockout cells. (*C*) Microsatellite analysis in FANCJ CRISPR knockout U2OS cells. MSI in FANCJ knockout U2OS cells were analyzed after aphidicolin (Aph) treatment. Microsatellite status at the indicated loci is indicated as stable (−) or unstable (+). Compared with untreated samples, changes in the banding pattern by aphidicolin treatment are shown as two plus signs (++). (*D*) MSI in FA-J patient lymphoblastoid cell lines (LCLs). Twelve microsatellites in three FA-J patient LCLs and two unaffected parent LCLs were analyzed by PCR-based MSI assay using primers labeled with a fluorescent dye. Unstable microsatellites in FA-J LCLs relative to unaffected LCLs are indicated as plus signs (+), and stable microsatellite are shown as minus signs (−). (*E*) Model of the maintenance of microsatellite stability by the FANCJ helicase.

To determine whether disruption of human FANCJ gives rise to MSI, we generated a FANCJ knockout U2OS cell line by genome editing with the CRISPR–Cas9 system. Guide RNAs targeting exon 5 of *Fancj* resulted in a knockout line devoid of detectable FANCJ by Western blotting (Fig. 7A). The integrity of 10 different microsatellites was assessed in the isogenic U2OS *Fancj*^+/+^ and *Fancj*^−/−^ cell lines. Consistent with the MSI observed in *Fancj*^−/−^ MEFs, U2OS *Fancj*^−/−^ cells, but not isogenic wild-type controls, exhibited instability associated with six out of 10 microsatellites (Fig. 7B,C).

Prompted by our findings that the replication inhibitor aphidicolin induces DNA damage, replication fork stalling/collapse, and sensitivity to *Fancj*-deficient MEFs, we considered the possibility that this phenotype may reflect increased problems at microsatellite sequences. To this end, we assessed the integrity of 10 microsatellites in isogenic U2OS *Fancj*^+/+^and *Fancj*^−/−^ cell lines following treatment with 0.2 µM aphidicolin for 24 h. Consistently, we observed that the extent of MSI in U2OS *Fancj*^−/−^ cells was exacerbated by aphidicolin treatment, but no such changes occurred in the controls (Fig. 7C).

To determine whether the MSI is also evident in human FA patients harboring mutations in FANCJ, we obtained primary lymphoblastoid cell lines (LCLs) from affected and unaffected members of a second-degree consanguineous FA-J family (two unaffected parents and three affected children), with affected individuals carrying a large autozygous region on chromosome 17, including FANCJ/BRIP1, and homozygous for the missense mutation c.1878A>T (exon 13 of FANCJ) with the effect p.E626D, which destabilizes the protein. Of the 12 microsatellites examined, no evidence of MSI was observed in the unaffected parents, whereas all affected children presented with MSI (Fig. 7D). Hence, loss of FANCJ in mouse or human cells gives rise to spontaneous MSI, which is exacerbated under conditions of replication stress.

## Discussion

Copy number changes within specific microsatellites have been implicated in >30 human CNS disorders. MSI is also a hallmark of certain cancers and is used as a diagnostic marker of Lynch syndrome, which is caused by germline mutations in MMR genes (Li 2008; Boland and Goel 2010). While MMR suppresses MSI by correcting base–base mismatches arising from strand slippages that occur at high frequency within microsatellite repeats, activities that prevent or dismantle DNA secondary structures that stabilize strand slippage events were unknown. Here, we establish a crucial role for the helicase FANCJ in preventing MSI in vertebrate cells, which is distinct from its role in the FA pathway. In the absence of murine FANCJ single-repeat contractions, four- to six-repeat expansions and more complex alterations arise, which is consistent with increased template slippage events at microsatellites (Boyer et al. 2013).

Our analysis of *Fancj*-deficient mice uncovered striking phenotypic similarities to other FA mouse models, including gonadal atrophy and subfertility, epithelial tumor predisposition, and cellular hypersensitivity to ICL-inducing agents (Parmar et al. 2009). Consistent with previous observations (Levitus et al. 2004; Litman et al. 2005), murine FANCJ is dispensable for the monoubiquitylation of FANCD2 and its subsequent recruitment into chromatin-associated repair foci. FANCJ also appears to be dispensable for the initial processing of the ICL lesion as DSBs accumulate in *Fancj*^−/−^ MEFs following treatment with MMC. Nevertheless, this observation does not exclude the possibility that ICL lesions are inappropriately processed in the absence of FANCJ, which could potentially give rise to erroneous repair and radial chromosome formation. While the strand exchange protein Rad51 accumulates in repair foci, DSBs persist in the absence of FANCJ, suggestive of a defect after ICL incision and downstream from Rad51 loading onto damaged chromatin.

Several unexpected phenotypes of *Fancj*^−/−^ mice absent from other mouse models of FA prompted us to investigate potential functions for FANCJ in the metabolism of G4-DNA and other DNA secondary structures. These phenotypic differences included (1) the absence of growth retardation or microphthalmia; (2) *Fancj^−/−^Fancd2*^−/−^ double-mutant mice and cell lines exhibit a more severe phenotype than either single mutant in some assays, including gonadal atrophy and cellular sensitivity to MMC; and (3) *Fancj*^−/−^ mice display increased predisposition to lymphomas, and cells derived from these mice are acutely sensitive to aphidicolin. Since FANCJ has been implicated in the metabolism of G4-DNA structures in chicken DT40 cells (Sarkies et al. 2012; Schwab et al. 2013) and *X. laevis* cell-free egg extracts (Castillo Bosch et al. 2014), it is perhaps surprising that *Fancj*^−/−^ MEFs are insensitive to G4-stabilizing drugs and do not present with telomere fragility. In contrast, cells deficient for the related helicase,*Rtel1*, which is also capable of unwinding G4-DNA structures in vitro, are hypersensitive to TMPyP4 and telomestatin and present with telomere fragility (Uringa et al. 2012; Vannier et al. 2012). In addition, *Rtel1^−/−^Fancj*^−/−^ double-knockout cells do not show heightened telomere fragility, yet, analogous to the phenotype of *dog-1; rtel-1* double mutants in *C. elegans* (Barber et al. 2008), double-knockout MEFs display enhanced levels of genome instability and accelerated senescence when compared with single mutants. These data suggest that, in mice, RTEL1 plays a prominent role in the metabolism of G4-DNA structures, while FANCJ plays no significant role in the metabolism of G4-DNA structures.

Our finding that loss of either murine or human FANCJ results in MSI is intriguing in light of previous observations. Loss of MMR in mouse models is also associated with increased predisposition to lymphoma (Li 2008), albeit with an earlier age of onset relative to *Fancj*^−/−^ mice. Several studies have reported that FANCJ interacts with the MMR protein MLH1 (Cannavo et al. 2007; Peng et al. 2007), yet the biological importance of this interaction and its importance for maintenance of microsatellites remain uncertain. Notably, *Mlh1* deficiency also gives rise to MSI, which is exacerbated by replication inhibition, and its mutation in humans results in Lynch syndrome and associated cancers (Lindblom et al. 1993; Bronner et al. 1994; Papadopoulos et al. 1994). Strikingly, the L607H mutation in MLH1 observed in hereditary nonpolyposis colon cancer (HNPCC) disrupts the interaction with FANCJ (Xie et al. 2010a), which raises the possibility that the FANCJ–MLH1 interaction is important for preventing colorectal cancers. Consistent with this supposition, mutations in FANCJ have been previously associated with rectal cancers (Rafnar et al. 2011). Despite these observations, FANCJ is not known to be mutated in Lynch syndrome, which is associated with MMR deficiency and strikingly elevated mutation rates, including, but not limited to, MSI. A potential explanation for this is that FANCJ, while playing a role in the maintenance of microsatellites, is not required for MMR per se, as its loss does not give rise to a spectrum of mutagenesis similar to that seen in the absence of MMR, which reflects a defect in the correction of base–base mismatches and other discontinuities in the double helix. Nevertheless, the MSI associated with loss of FANCJ could explain its impact in colorectal and pancreatic cancers. In addition, as human *Fancj* heterozygous and homozygous mutations are associated with some types of cancers, the co-occurrence of MSI might suggest that these tumors are ICL-sensitive.

Current models propose that expansions or contractions of repetitive sequences arise as a result of strand slippage during DNA polymerization, which is favored by the propensity of microsatellites to form DNA secondary structures within ssDNA that stabilize strand misalignments (Wang and Vasquez 2014). Indeed, the enhanced MSI observed following treatment with replication inhibitors in *Fancj*-deficient cells could be explained by the fact that this increases the presence of ssDNA at the replication fork, which favors DNA secondary structure formation. The impact of replication inhibitors on MSI provides a plausible explanation for the hypersensitivity of *Fancj*^−/−^ MEFs to aphidicolin, which is associated with increased fork asymmetry/stalling, reduced fork extension rates, and reduced interorigin distance. The latter presumably reflects the consequences of fork stalling at microsatellite sequences. Importantly, of the microsatellites that are unstable in *Fancj*^−/−^ MEFs, CAG/CTG, TTC/GAA, CA/TG, and CT/AG repeats are potential DNA slippage sites, and CAG/CTG, TTC/GAA, CT/AG, and CA/TG are believed to form hairpin-like structures, triplex structures, triplex structures, and Z-DNA structures, respectively (Zhao et al. 2010). Since FANCJ has been shown to unwind a variety of DNA secondary structures in vitro (Brosh and Cantor 2014), including, but not limited to, hairpins and triplex structures, we propose that FANCJ acts to suppress MSI by dismantling DNA secondary structures that favor repeat sequence expansion or contraction via a strand slippage mechanism (Fig. 7E), which is distinct from error correction by MMR.

## Materials and methods

*Fancj* knockout mice were generated by a gene trap strategy. Primary MEFs were derived at 13.5 d post-coitum. For immortalization, primary MEFs were transfected with large T SV40 plasmid. For histology and post-mortem tissues, samples were fixed, paraffin-embedded, sectioned, and stained with hematoxylin and eosin. For immunohistochemistry, tissue sections were stained by avidin–biotin complex methods. For FANCJ expression and FANCD2 ubiquitylation, cell lysates were prepared from *Fancj*^+/+^ and *Fancj*^−/−^ MEFs and U2OS cells and subjected to Western blot with the indicated antibodies. For clonogenic survival assays, MEFs were seeded in 10-cm dishes and stained with crystal violet 7–13 d after treatment with DNA-damaging agents. For immunofluorescence staining experiments, MEFs were permeabilized, fixed, and stained with the indicated antibodies. For chromatin fractionation, insoluble chromatin fractions and soluble fractions were separated by centrifugation. For PFGE experiments, cells were embedded in agarose plugs and treated with proteinase K overnight. Genomic DNA was separated by PFGE apparatus and stained with ethidium bromide. For SA-β-gal assay, primary MEFs were stained using senescence cell histochemical staining kit (Sigma). For DNA combing, primary MEFs were pulse-labelled with IdU for 20 min and subsequently labelled with CldU for 20 min. Extracted DNA was stretched on silanized coverslips and stained with two different anti-BrdU antibodies. For telomere FISH experiments, swollen cells were fixed by methanol and acetate solution and dropped on a glass slide. Slides were hybridized with PNA telomeric probe. To generate human FANCJ knockout U2OS cells by the CRISPR/Cas9 system, FANCJ guide oligos were cloned into pX462 plasmid and used for transfection. Transfected clones were isolated by limiting dilution and subjected to Western blot and MSI analysis. For MSI analysis, microsatellites were amplified by PCR with fluorescent-labelled primers or nonlabelled primers. Labelled PCR products were analyzed by ABI3130XL systems. Nonlabelled PCR products were analyzed by polyacrylamide gel electrophoresis. For sequencing microsatellites, PCR products were cloned into empty vector and used for transformation. Plasmids were prepared and sequenced from >20 bacterial clones. See also the expanded Materials and Methods section in the Supplemental Material for further details.

## Supplementary Material

Supplemental Material
