# Supplementary material for: FANCJ suppresses microsatellite instability and lymphomagenesis independent of the Fanconi anemia pathway
Source: Genes Dev. 2015 Dec 15;29(24):2532–46. doi: 10.1101/gad.272740.115 (PMC4699383; doi:10.1101/gad.272740.115)
Supplement: Supplemental Material [file supp_29_24_2532__index.html]

FANCJ suppresses microsatellite instability and lymphomagenesis independent of the Fanconi anemia pathway — Supplemental Material 

# FANCJ suppresses microsatellite instability and lymphomagenesis independent of the Fanconi anemia pathway

## Supplemental Material

**Files in this Data Supplement:**

- Supp Material.docx
