# Supplementary material for: FANCJ suppresses microsatellite instability and lymphomagenesis independent of the Fanconi anemia pathway
Source: Genes Dev. 2015 Dec 15;29(24):2532–46. doi: 10.1101/gad.272740.115 (PMC4699383; doi:10.1101/gad.272740.115)
Supplement: Supplemental Material [file supp_gad.272740.115_SuppMaterial.docx]

**Supplemental Information**

**FANCJ suppresses microsatellite instability and lymphomagenesis**

**independent of the Fanconi Anemia Pathway**

Kenichiro Matsuzaki^1^, Valerie Borel^1^, Carrie A. Adelman^1^, Detlev Schindler^2^

& Simon J. Boulton^1*^

^1^ DNA Damage Response laboratory, The Francis Crick Institute, Clare Hall Laboratories, South Mimms, EN6 3LD, UK.

^2^ Department of Human Genetics, Biozentrum, University of Wurzburg, Germany.

*Correspondence to: simon.boulton@crick.ac.uk

**This PDF file includes:**

Supplemental Figures 1-4

Extended Materials and Methods

**
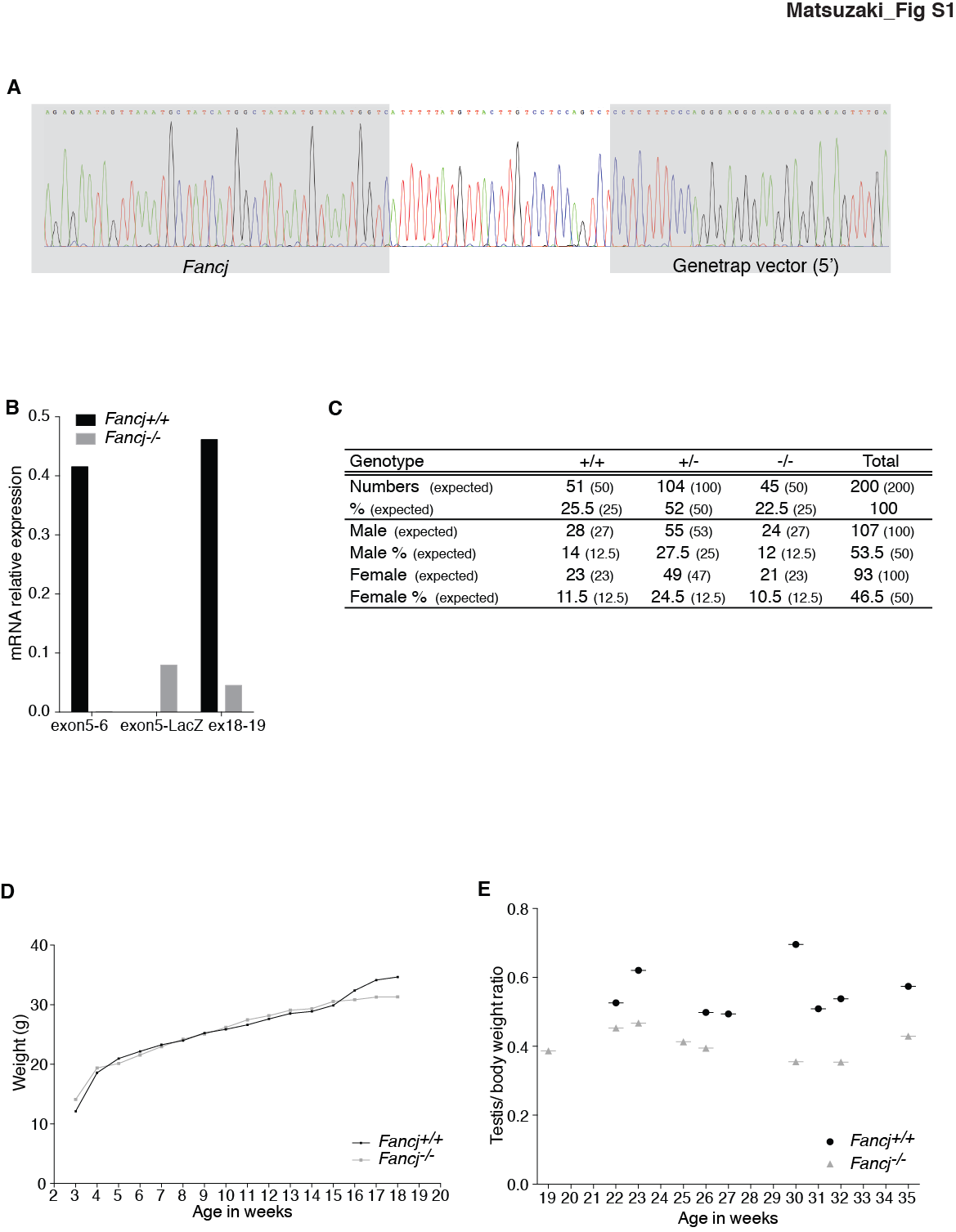
**

**Figure S1:**

(**A**) Genetrap vector insertion in Fancj genomic locus

(**B**) Fold reduction in *Fancj* transcript in *Fancj* mice relative to exon 3-4 expression analyzed by qRT-PCR using primers spanning the junction of exons 5 and 6 (Wild-type) or 5 and LacZ (Mutant) or 18 and 19.

(**C**) *Fancj* mice mendelian ratios.

(**D**) Weight analysis of *Fancj* mice. Error bars are not shown to render the graph readable; Data are from males and females with at least 5 mice measured at each time point.

(**E**) Testis weight over aging. (Significance: Pearson r test, p=0.75).

**
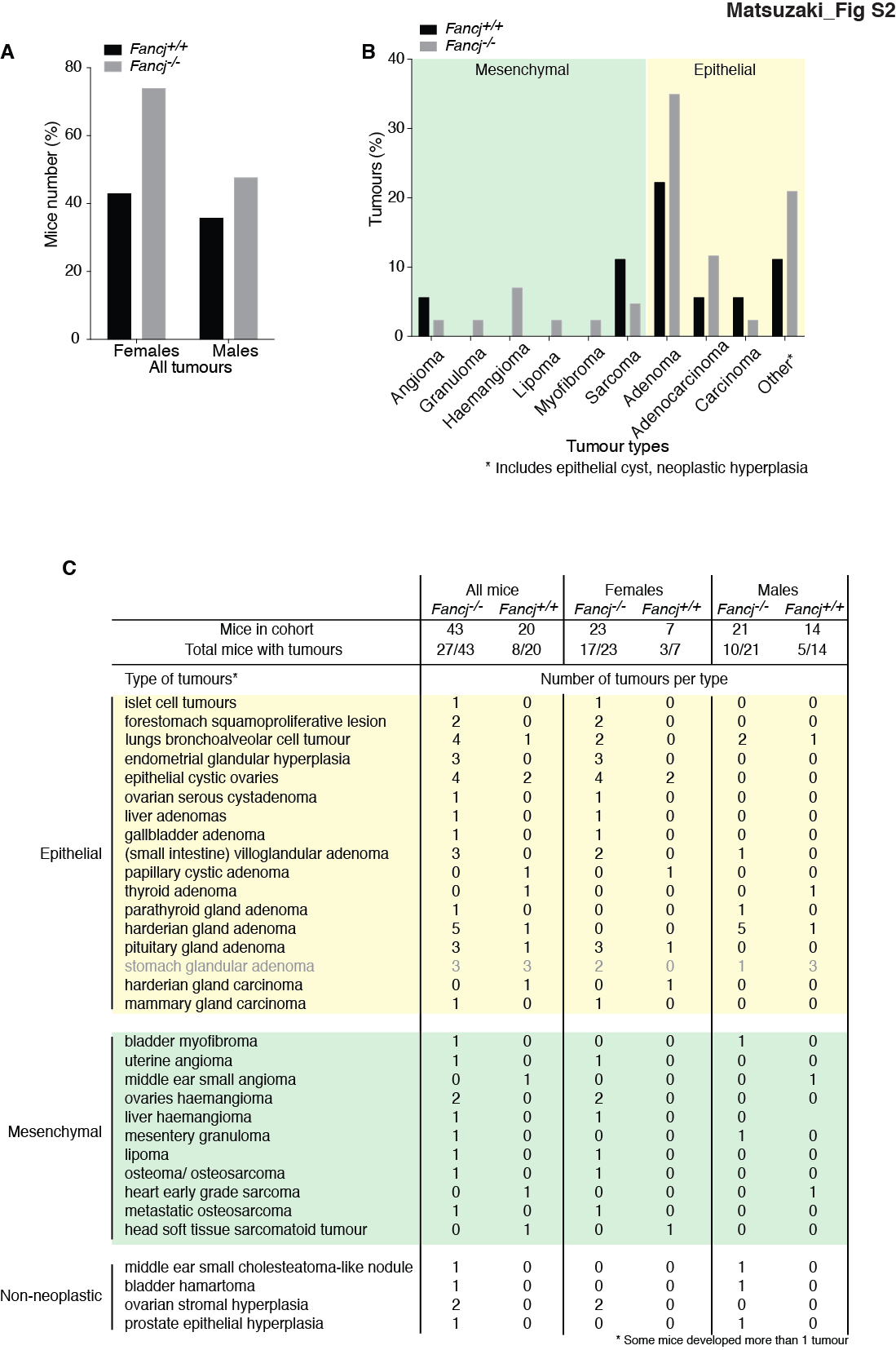
**

**Figure S2:** **Tumour spectrum observed in *Fancj* knockout mice**.

(**A**) Frequency of tumours in *Fancj* females and males. (Significance: Fisher’s exact test; males and females, p=0.2).

(**B**) Frequency of tumours by type.

(**C**) Frequency and tumour number and spectrum in all mice, females only and males only. Grey text corresponds to tumour specific to mouse background.

**
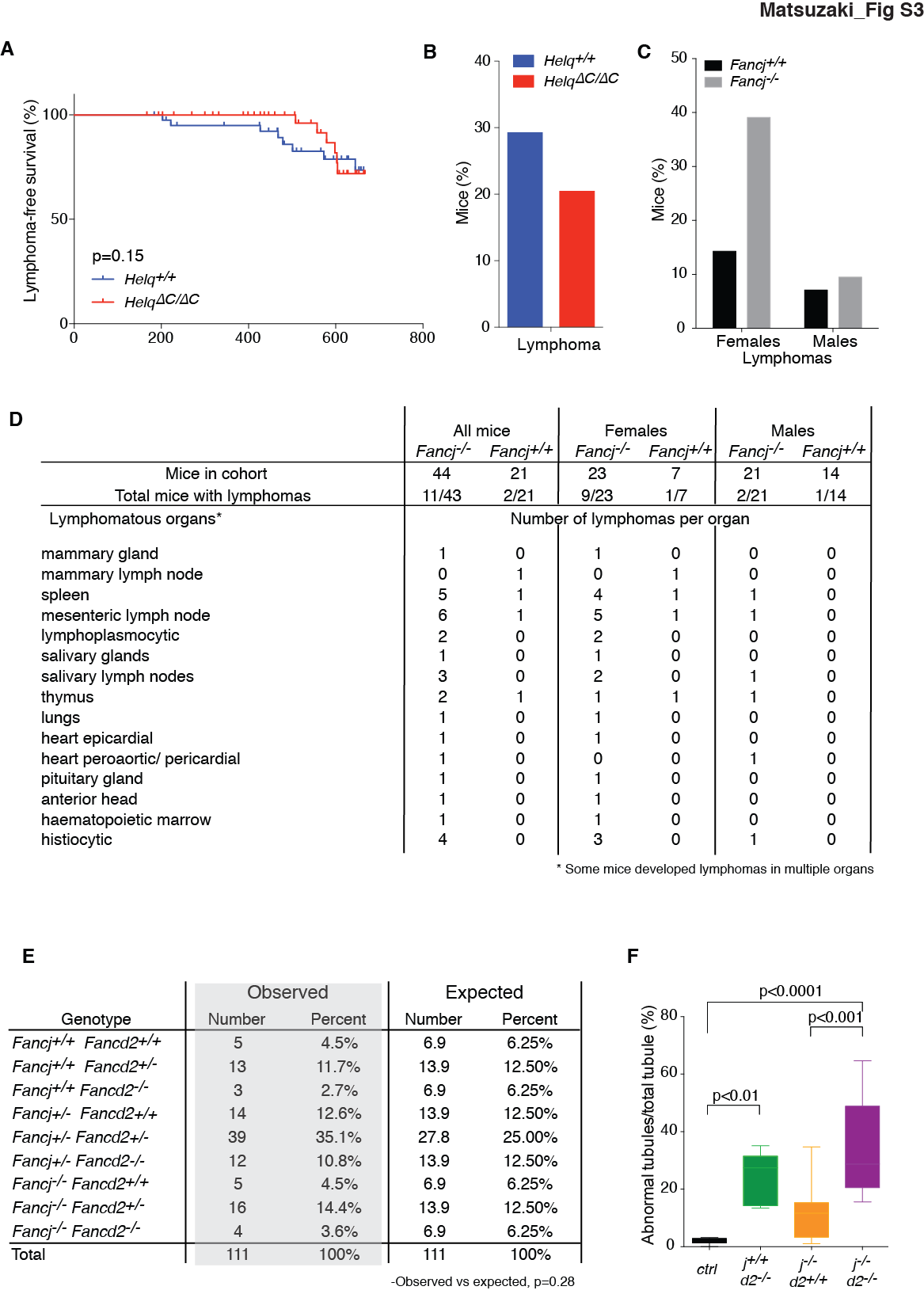
**

**Figure S3:** ***Fancj* knockout mice exhibit phenotypes distinct from the canonical Fanconi Anemia phenotype**

(**A**) Lymphoma-free survival of *Helq* mice (Significance: Mantel-Cox test, p=0.15; n=41 *Helq^∆C/∆C^,* N=44 *Helq^+/+^*).

(**B**) Frequency of *Helq* mice with lymphomas. (Significance: Fisher’s exact test, p=0.18).

(**C**) Frequency of lymphomas in *Fancj* females and males. (Significance: Fisher’s exact test, females: p=0.1; males: p=0.4).

(**D**) Frequency and lymphoma number and spectrum in all mice, females only and males only.

(**E**) *Fancj/Fancd2* mice mendelian ratios. Double heterozygous were used to compile these data. (Significance: Chi-square test of observed vs expected, p=0.28).

(**F**) Abnormal seminiferous tubules quantification on *Fancj/Fancd2* testis sections. (Significance: one-way ANOVA, p<0.0001).

**
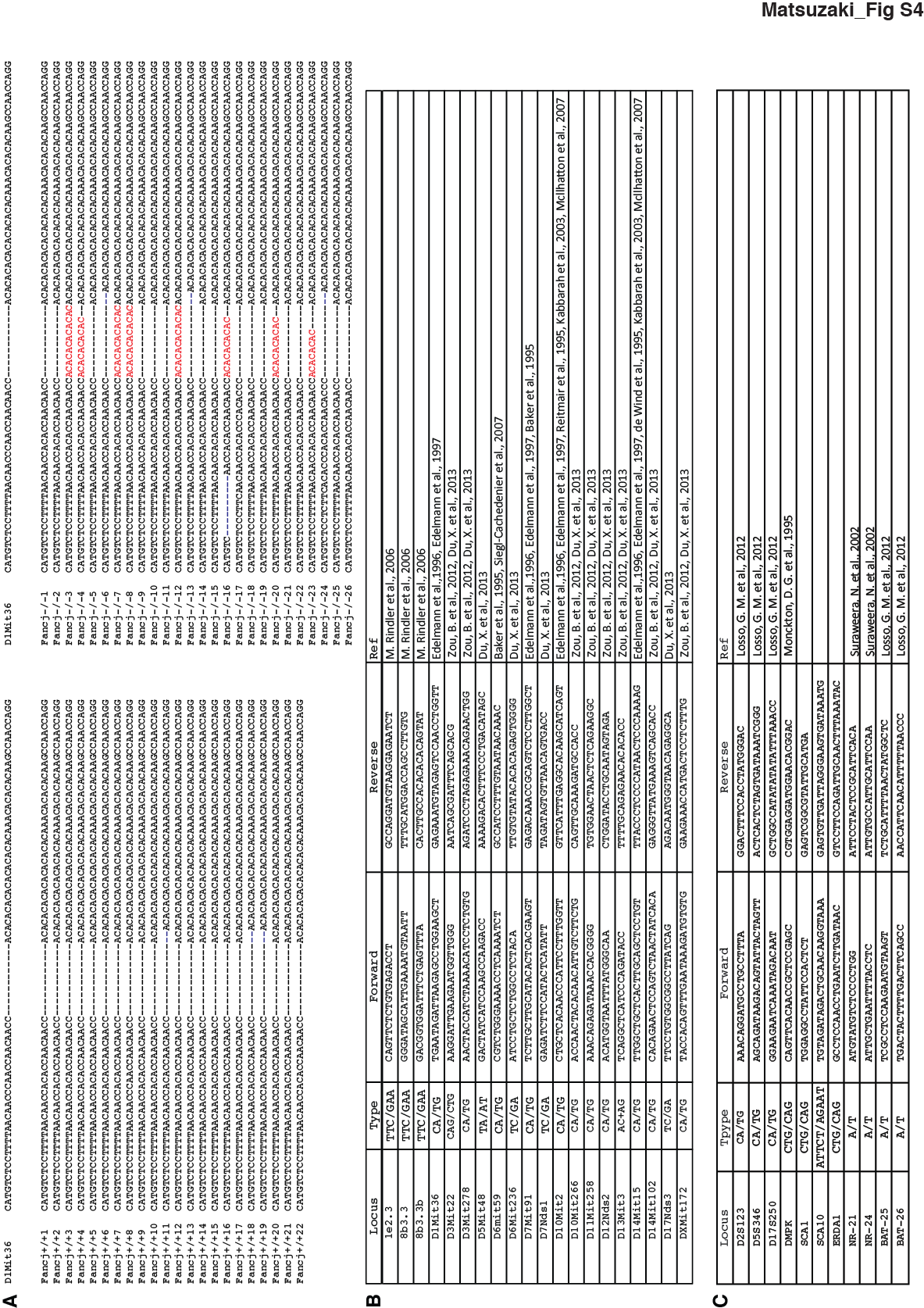
**

**Figure S4:** **Sequence analysis of D1Mit36 in *Fancj* knockout cells**

(**A**) Characterization of D1Mit36 microsatellite sequence in *Fancj+/+* (left) and *Fancj-/-* (right) MEFs. PCR products of D1Mit36 were cloned into pBluescriptKS+ vector and used for transformation. Plasmids were prepared from 22 colonies (+/+) and 26 colonies (-/-) and sequenced. Inserted and deleted sequences were indicated by red and blue, individually.

(**B**) Primers used for mouse microsatellite analysis.

(**C**) Primers used for human microsatellite analysis.


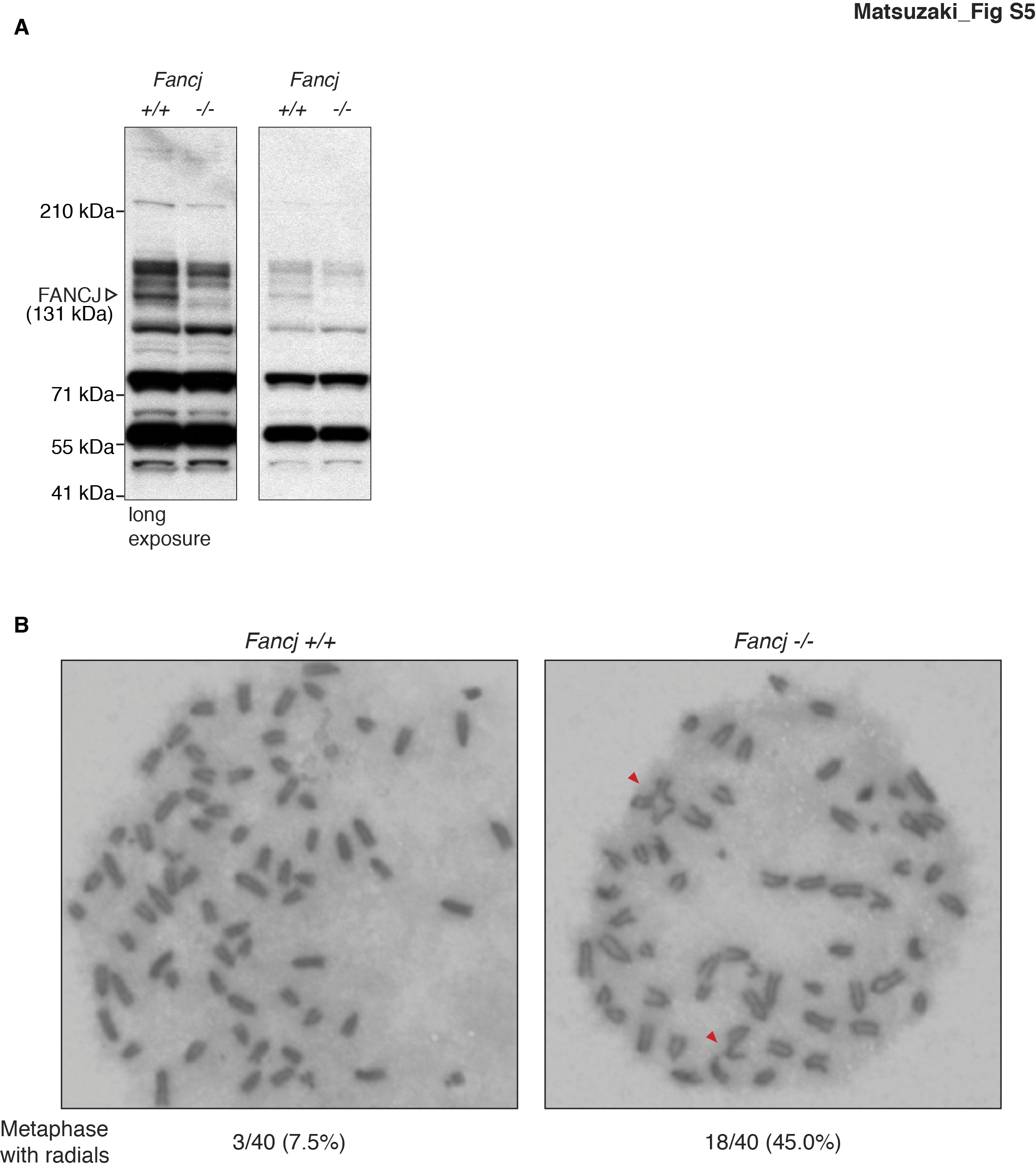


**Figure S5:** **Metaphase chromosomes with radials in *Fancj* knockout MEFs**

(**A**) Western-blot for FANCJ in *Fancj^+/+^* and *Fancj^-/-^* MEFs. Arrow indicates FANCJ protein. Left, long exposure. Right, short exposure. (**B**) Frequency of metaphase with radial chromosomes in *Fancj^+/+^* (left) and *Fancj^-/-^* (right) MEFs treated with 5ng/ml MMC for 16h.

**Supplemental Materials and Methods**

**Animals**

Mice deficient for FANCJ were generated using an ES cell line Brip1^Gt(RRI409)Byg^ available from Baygenomics (University of California, Davis) in which a genetrap vector pGT0LxF containing a β-Geo cassette was inserted between exon 5 and 6. The precise localisation of the genetrap vector has been determined by primer walk on intron 5 of the Fancj gene using one primer located in the genetrap vector sequence (EN2Intr, 5’-GGCCTGCTCAAACCTGAACC-3’) and primers located every 500bp in intron 5.

Brip1^Gt(RRI409)Byg^  ES cells were injected into C57BL/6Jax host blastocysts and implanted into pseudopregnant females. Chimeric mice were obtained and bred to SV129 mice. The resulting heterozygous (*Fancj^+/-^*) mice were bred to obtain homozygous *Fancj^-/-^*. Genotyping of the offspring was confirmed by western-blot, qRT-PCR (Exon3, 5’-GTCCCACAGGAAGTGGAAAA-3’; Exon4, 5’-GTGGTGCCTCAGGCTTTTTA-3’; Exon18, 5’-GGACGATCGCTTCAATAACAA-3’; Exon19, 5’-GAGAACTCGGTCAGCGACTC-3’; LacZ, 5’-GGCCTCTTCGCTATTACGC-3’) as well as PCR using the following primers (FancJ-Ex5-1-S, 5’-TGCCAAGAAACAGGCATCTATAC-3’; FancJ-Ex5-501-1000-AS, 5’-ATGACCTCTTCTGATCTCTGCTG-3’; EN2intr, 5’-GGCCTGCTCAAACCTGAACC-3’).

For longevity studies, mice were allowed to age and observed for development of disease. The endpoint of the study was set at 21 months but if they appeared unhealthy or got palpable tumours beforehand, animals were sacrificed. They were then subjected to full necropsy.

For blood sampling, mice were placed in a heating chamber for 10 minutes and then tail prick was performed and blood was collected into EDTA coated capillaries. 10µl of blood was used to perform a full blood differential on the ABCplus Vet blood analyser (Horiba).

All animal experimentations were undertaken in compliance with UK Home Office legislation under the Animals (Scientific Procedures) Act 1986.

**Histology, immunohistochemistry**

For histology and post-mortem tissues, samples were fixed in 10% Neutral buffered formalin (NBF), paraffin embedded, sectioned at 4µm and stained with haematoxylin and eosin.

For immunohistochemistry, samples were prepared using standard methods. In brief, tissue sections were processed for staining by microwaving in 0.01M citrate buffer, pH 6. After incubation with primary antibodies (PLZF, Santa Cruz sc28319; Sox9, Millipore AB5535), samples were incubated with biotinylated secondary antibody (Vector) followed by incubation with Avidin Biotin Complex (Vector); slides were developed in 3,3’-diaminobenzidine (DAB) substrate (Vector) and counterstained in haematoxylin.

Testes, ovaries and liver images were acquired at 20X using an Axio Scan.Z1 (Zeiss) and stainings were quantified using ImageJ software on serial sections and tubules with similar diameter. Tumour and lymphomas images were taken using a Nikon Digital Sight DS-Ri1 camera paired to a Nikon 90i Eclipse microscope. Imaging software was NIS-Elements AR Ver 4.0, 64bit.

**Cell line derivation**

Mouse embryonic fibroblasts (MEFs) have been derived at 13.5dpc using standard protocol and cultured in Dulbecco's modified Eagle's medium (DMEM) (Invitrogen) supplemented with 15% fetal bovine serum and 1% penicillin-streptomycin (Invitrogen). MEFs immortalized by Large T-SV40 were maintained with 10% FBS.

**Statistical analysis**

GraphPad Prism was used for all statistical analysis: Kaplan–Meier plots for survival and calculate significance using Log-rank (Mantel–Cox) test, unpaired t-test for staining quantification statistics, Tukey’s multiple comparisons test for *Fancj/Fancd2* experiments analysis.

**Antibodies**

Antibodies used for western blot and immunofluorescence staining were FANCJ (Novus, NBP1-31883), FANCD2 (Epitomics, 2986-1), Tubulin (Sigma T6199), Histone H3 (Abcam 10799), γ-H2AX (Millipore 05-636), and Rad51 (Santa Cruz SC-8364).

**Western blot**

Cell were harvested and resuspended in benzonase buffer (20mM Tris-HCl pH7.5, 40mM NaCl, 2mM MgCl2, 0.5% NP-40, 50U/ml benzonase, 1x Protease inhibitor, 1x phosphatase inhibitor) and incubated at 4°C for 10 min. NaCl was added to the sample at a final concentration of 450mM. After 30min incubation, cell lysates were clarified by centrifugation. Samples were separated by NuPAGE 4-12% Bis-Tris gels or 3-8% Tris-acetate gels and transferred onto a PVDF membrane. The blots were blocked with 5% milk in PBST for 30min, and incubated with primary antibody and HRP-conjugated secondary antibody.

**Senescence-associated β-galactosidase**

For senescence-associated β-galactosidase (SA-β-gal), primary MEFs were plated in triplicate on 6-well plates. Cells were stained using Senescence cells histochemical staining kit (sigma) according to the manufacturer’s protocol.

**Clonogenic survival**

SV40 immortalized MEFs were plated in triplicate on 10cm-dishes. Eight hours after seeding, cells were treated with mitomycin C, camptothecin, aphidicolin, TMPyP4, telomestatin, and pyridostatin for 18-60 hours. For UV irradiation, cells were exposed to the indicated dose of UV. After 8-13 days, cells were fixed and stained in 4% crystal violet / 20% ethanol. The number of colonies were counted and normalized for plating efficiency.

**Pulse field gel electrophoresis**

Pulse field gel electrophoresis was performed as previously described (Adelman et al. 2013). SV40 immortalized MEFs were treated with 1µg/ml MMC for 1hour. Cell were incubated in MMC-free media and harvested at indicated time points.

**DNA combing**

DNA combing was performed as previously(Vannier et al. 2013). Briefly, primary MEFs were pulse-labeled with 20µM IdU for 20min at 37°C and subsequently labelled with CldU at 37°C for 20min. labelled DNA were extracted in agarose plugs. Extracted DNA was stretched on silanized coverslips and denatured in 2x SSC/50% Formamide at 75°C for 2 min. After dehydration, coverslips were blocked in 1% blocking reagent/PBS (Roche) at 37°C for 60min. IdU strands and CldU strands were detected by Rat anti-BrdU antibody (AbD Serotec) and mouse anti-BrdU antibody (BD Biosciences) individually.

**MSI analysis**

For mouse microsatellite analysis, we analyzed *Fancj^+/+^* and *Fancj^-/-^* MEFs generated from same litter at the same passage to eliminate background differences. After genomic DNA preparation, microsatellites were amplified by PCR with the primers indicated in Figure S4B. PCR products were separated by 6% polyacrylamide gel and stained with ethidium bromide. Samples showing different band pattern from wild type are designated the microsatellite instability positive (+). Among microsatellite instability positive, samples showing additional higher and lower molecular weight bands were classified as the expansion and the contraction, individually. We used three independently generated sets of *Fancj^+/+^* and *Fancj^-/-^* MEFs for mouse MSI analysis.

1^st^ Set: Fancj^+/+^, Fancj^-/-^ #1

2^nd^ Set: Fancj^+/+^, Fancj^-/-^ #1, Fancj^-/-^ #2

3^rd^ Set: Fancj^+/+^, Fancj^-/-^ #1, Fancj^-/-^ #2

For the sequence analysis of PCR products, PCR products were inserted the pBluescript vector and transformed in to bacteria. Plasmids were prepared from more than 20 colonies and sequenced.

For human microsatellite analysis, microsatellites were amplified by PCR with MSI analysis kit (Promega) and the primers indicated in Figure S4C. PCR products were analysed by polyacrylamide gel electrophoresis. In addition to PAGE analysis, microsatellite analysis was performed using fluorescent dye-labeled primes on an ABI 3130XL system, followed by analysis using GeneMapper and Peak Scanner softwares (Applied Biosystems).

**Immunofluorescence staining**

MEFs were plated and cultured on coverslips. Cells were permeabilized with IF CSK buffer (10mM PIPES pH 6.8, 100mM NaCl, 300mM Sucrose, 3mM MgCl2, 1mM EGTA, 0.5% Triton X-100, 1x protease inhibitor cocktail, 1x phosphatase inhibitor) and fixed with 2% PFA at room temperature for 15 min. Coverslips were blocked in PBS with 3% BSA and Triton X-100 at room temperature for 30 min. Coverslips were incubated in blocking buffer with primary antibody at 4ºC overnight. After washing in PBS with 0.1% Triton X-100, coverslips were incubated with Alexa Fluor 488 or 594 secondary antibody at room temperature for 1 hour. Coverslips were treated with DAPI and mounted with Vectashield media.

**Metaphase spread and Telomere FISH**

Metaphase spread and telomere FISH were performed as previously described (Vannier et al. 2012). MEFs were treated with colcemid for 90min. After harvesting, cells were pelleted and 5ml of 0.075M KCl was added dropwise to swell cells. After 15min incubation, fixation buffer (MeOH: AcOH ; 3:1) was added to the cell suspension. Cells were washed and resuspended in fixation buffer. The cell suspension was dropped on a glass slide and air dried. Slides were hybridized with PNA telomeric probes (Bio Synthesis) and DNA was stained with DAPI.

**Chromatin fractionation**

For chromatin fractionation, cells were resuspended in CSK buffer (10mM HEPES pH7.9, 150mM NaCl, 300mM sucrose, 1mM MgCl2, 1mM EDTA, 0.2% Triton X-100, 1x protease inhibitor, 1x phosphatase inhibitor) and incubated on ice for 10min. this cell suspension was designated the whole cell extract. Chromatin-containing fractions were pelleted. Supernatant was designated the soluble fraction. The pelleted chromatin fractions were washed in CSK buffer and resuspended in nuclease digestion buffer. The samples were sonicated using Bioruptor and treated with benzonase at 37°C for 30min. This fraction was designated the chromatin fraction.

**Generation of human FANCJ knockout cell lines by CRISPR/Cas9 systems**

Human FANCJ knockout cell lines were generated following the double Cas9-nicking strategy protocol described previously (Ran et al. 2013). We designed a pair of sgRNAs targeting human FANCJ exon5 using CRISPR Design Tool (http://tools.genome-engineering.org). The following sgRNA sequences were used: hFANCJ guide4 (AGATAACTTTGCAGCCAGAG) and hFANCJ guide6 (AAAGTTATCTGCTAAGAAAC). This pair of guide RNAs induces a nick to opposite strand of the target site to minimize off-target activity. Guide oligos were annealed and ligated into pX462 plasmid. Using 4D-Nucleofector system (Lonza), U2OS cell were transfected with pX462 plasmids containing FANCJ guide sequence. After transfection, single cells were isolated by limiting dilutions. Cells were propagated and subjected to western blot to verify FANCJ protein expression.

**References:**

Adelman CA, Lolo RL, Birkbak NJ, Murina O, Matsuzaki K, Horejsi Z, Parmar K, Borel V, Skehel JM, Stamp G et al. 2013. HELQ promotes RAD51 paralogue-dependent repair to avert germ cell loss and tumorigenesis. *Nature* **502**: 381-384.

Ran FA, Hsu PD, Wright J, Agarwala V, Scott DA, Zhang F. 2013. Genome engineering using the CRISPR-Cas9 system. *Nature protocols* **8**: 2281-2308.

Vannier JB, Pavicic-Kaltenbrunner V, Petalcorin MI, Ding H, Boulton SJ. 2012. RTEL1 dismantles T loops and counteracts telomeric G4-DNA to maintain telomere integrity. *Cell* **149**: 795-806.

Vannier JB, Sandhu S, Petalcorin MI, Wu X, Nabi Z, Ding H, Boulton SJ. 2013. RTEL1 is a replisome-associated helicase that promotes telomere and genome-wide replication. *Science* **342**: 239-242.
